# Non-communicable diseases in South Africa: The role of primary care

**DOI:** 10.4102/safp.v68i1.6302

**Published:** 2026-06-02

**Authors:** Indiran Govender, Ramprakash Kaswa

**Affiliations:** 1 Department Family Medicine and Primary Health Care, Faculty of Health Sciences, Walter Sisulu University, Mthatha, South Africa; 2 School of Rural Medicine, Charles Sturt University, New South Wales, Australia

After three decades of democratic governance, South Africa is experiencing a significant health transition characterised by a quadruple burden of diseases, including communicable diseases, non-communicable diseases (NCDs), perinatal and maternal disorders, and injury-related conditions.^1,2^ Relating to this burden of disease, previous editorials have discussed the problems of road traffic accidents, obesity and gender-based violence in South Africa.^3,4,5^ The increasing prevalence of NCDs poses a growing challenge to healthcare systems. As people live longer and NCDs rise, urgent interventions are required to reduce and manage this epidemic.^6^ Non-communicable diseases are emerging in both rural and urban areas, most notably among impoverished populations living in urban settings, thereby placing additional pressure on both acute and chronic healthcare services. The global trend indicates a rise in these health issues, a phenomenon that is notably more severe within the South African context. The increasing prevalence of NCDs significantly contributes to premature mortality.^1,7^

About two billion people live with NCDs, which encompass cardiovascular diseases, diabetes mellitus, cancers, chronic respiratory diseases, and mental health conditions. The global health equity crisis caused by NCDs is so vast that deciding how to respond can seem overwhelming.^4,6,8^ However, the best starting point is primary healthcare (PHC). The PHC approach is a globally scalable, holistic method that can reduce the burden of NCDs and improve the health of billions.

The South African National Strategic Plan for the prevention and control of NCDs adopts a 90-60-50 framework. According to this, 90% of adults should be aware of their NCD status, 60% should receive intervention, and 50% should maintain control.^1^ A comprehensive PHC approach using the 5 × 5 strategy targets five major NCDs: cardiovascular diseases, cancer, chronic respiratory diseases, diabetes, and mental health, as well as five common risk factors: tobacco use, unhealthy diet, physical inactivity, harmful alcohol consumption, and climate change.^1,7^ The strategy emphasises integrated interventions to address these prioritised health issues through a PHC approach.

The South African Ministry of Health has amended the laws under *Act No. 52 of 1972* to regulate sugar and salt levels in all foods and to require nutritional labels for fats, carbohydrates, and other nutrients. This governmental initiative has become a global trend and has achieved success in several countries. The regulations also recommended classifying healthy foods, defining criteria for health claims, and implementing mandatory front-of-pack labels with recognisable logos.^9^ These labels will help consumers identify foods that exceed limits for added sugar, sodium, and saturated fats, thereby addressing the rising issues of obesity, NCDs, and associated mortality in South Africa. Such policies are crucial for mitigating the rising rate of NCDs.^8^ However, it is also essential to recognise that current monitoring and enforcement are inadequate, although efforts are underway to improve compliance.

People with NCDs often show no symptoms until the disease has advanced significantly, with the first signs sometimes being a heart attack or stroke.^10^ Screening asymptomatic individuals for key risk factors can help identify those at high risk and potentially prevent disease progression. Because primary care services are typically the first point of contact within the health system, they offer the best opportunity to identify high-risk individuals, even if they are visiting for unrelated health issues.^2,11^ Four key PHC elements are vital for addressing the NCDs epidemic across different settings: integrating services, implementing innovative delivery methods, prioritising people living with or at risk of NCDs, and leveraging the PHC approach.

Firstly, the PHC approach emphasises integrated, high-quality primary care services that offer individuals continuity, comprehensiveness and coordination of care from their first contact with the health system.^11^ Exercise and a healthy diet must be integrated within the individual’s context. Primary care providers build sustainable relationships with patients rather than delivering incidental, provider-led care. Primary healthcare workers should encourage healthy lifestyles at every visit and actively screen for and manage NCDs.^7,10^

Secondly, enhancing healthcare services by upgrading PHC facilities and ensuring access to essential medicines to improve the management of NCDs, as well as implementing context-relevant models of care that may involve task-shifting strategies through training primary care providers and teams to manage NCDs.^12^ Utilising trained community health workers for NCD prevention, health promotion, and follow-up care. Providing patient education and counselling as integral parts of communication and health literacy initiatives to empower patients.^10^

Thirdly, individuals living with NCDs, or those at risk of developing them, require long-term care that is proactive, patient-centred, community-based, and sustainable.^7^ This encompasses community programmes that screen for body mass index (BMI), blood pressure, blood glucose, and cholesterol levels. During these visits, immunisation status should be verified, and influenza, shingles, respiratory syncitial virus (RSV), and pneumococcal vaccines should be strongly recommended. As part of lifestyle modifications, emphasis should be placed on healthy eating, regular physical activity, smoking cessation, and the avoidance of harmful alcohol consumption. Such care can only be delivered equitably through the establishment of robust PHC systems.^1^

Finally, a notable aspect of the PHC approach is its distinctly multisectoral and prevention-focused perspective.^11^ As the rising prevalence of NCDs exceeds the capacity of health systems to respond effectively, addressing risk factors and health determinants becomes increasingly urgent, necessitating interventions that extend beyond the healthcare sector. Non-specialist healthcare providers can successfully implement a comprehensive risk approach at the PHC level. Therefore, PHC provides a practical, affordable, and equitable means of reaching populations in need of healthcare for NCDs.^6,10^

In conclusion, it is imperative that the government first recognise the challenge and demonstrate full commitment to addressing NCDs and allocating the resources to implement the national and provincial policies, such as the South African National Strategic Plan. Given their significant impact, the management of these conditions cannot depend solely on specialists or hospitals. The high volume of patients, which contributes to the overload of referral services, results in substantial costs to both the healthcare system and individuals. As people live longer, NCDs will continue to increase, and this will have multiple effects on healthcare delivery, morbidity and costs. The implementation of person-centred population-wide prevention and management strategies of NCDs, driven by primary care providers, is an urgent unmet need in our PHC system.
